# Modified-Chitosan/siRNA Nanoparticles Downregulate Cellular CDX2 Expression and Cross the Gastric Mucus Barrier

**DOI:** 10.1371/journal.pone.0099449

**Published:** 2014-06-12

**Authors:** Ana Sadio, Jenny K. Gustafsson, Bruno Pereira, Carla Pereira Gomes, Gunnar C. Hansson, Leonor David, Ana Paula Pêgo, Raquel Almeida

**Affiliations:** 1 IPATIMUP- Instituto de Patologia e Imunologia Molecular da Universidade do Porto, Porto, Portugal; 2 INEB – Instituto de Engenharia Biomédica, Universidade do Porto, Porto, Portugal; 3 Gastroenterology Department, Unidade Local Saúde da Guarda, Guarda, Portugal; 4 Gulbenkian Programme for Advanced Medical Education, Lisboa, Portugal; 5 Faculdade de Medicina da Universidade do Porto, Porto, Portugal; 6 Department of Medical Biochemistry, University of Gothenburg, Gothenburg, Sweden; 7 Faculdade de Engenharia da Universidade do Porto, Porto, Portugal; 8 ICBAS - Instituto de Ciências Biomédicas Abel Salazar, Universidade do Porto, Porto, Portugal; Centro Nacional de Investigaciones Oncológicas (CNIO), Spain

## Abstract

Development of effective non-viral vectors is of crucial importance in the implementation of RNA interference in clinical routine. The localized delivery of siRNAs to the gastrointestinal mucosa is highly desired but faces specific problems such as the stability in gastric acidity conditions and the presence of the mucus barrier. CDX2 is a transcription factor critical for intestinal differentiation being involved in the initiation and maintenance of gastrointestinal diseases. Specifically, it is the trigger of gastric intestinal metaplasia which is a precursor lesion of gastric cancer. Its expression is also altered in colorectal cancer, where it may constitute a lineage-survival oncogene. Our main objective was to develop a nanoparticle-delivery system of siRNA targeting CDX2 using modified chitosan as a vector. CDX2 expression was assessed in gastric carcinoma cell lines and nanoparticles behaviour in gastrointestinal mucus was tested in mouse explants. We show that imidazole-modified chitosan and trimethylchitosan/siRNA nanoparticles are able to downregulate CDX2 expression and overpass the gastric mucus layer but not colonic mucus. This system might constitute a potential therapeutic approach to treat CDX2-dependent gastric lesions.

## Introduction

Targeting transcription factors therapeutically remains a challenge, as they are not conventional “druggable” molecules, such as proteins with enzymatic activity that can be inhibited by small molecules or receptor proteins that can be targeted by antibodies [1], [2]. The discovery of RNA interference has revolutionized this field as, theoretically, any target can be hit with this strategy [3]. RNA interference consists of a double-stranded small interfering RNA (siRNA) with a length of about 20–30 nucleotides that leads to a sequence specific enzymatic cleavage of a target mRNA through complementary base pairing [4]–[6]. Although promising, the clinical application of siRNAs continues to face problems related to their effective cellular delivery. Therefore, the development of delivery systems that can protect and transport siRNA is a field of active research.

Chitosan (CH) is a polymer of β-1-4 N-acetylglucosamine and D-glucosamine residues derived by partial deacetylation of chitin. Since this is a natural, biocompatible, biodegradable, mucoadhesive and non-toxic polymer with a relative low-cost production, it has been broadly studied for the delivery of both plasmid DNA and siRNA due to its capacity, when positively charged, to protect nucleic acids from degradation by endonucleases [7]–[10]. Primary amine residues of CH are protonated at pH values below its pKa (∼6.5) giving it the capacity to complex anionic compounds, such as the phosphate groups of nucleic acids, enabling the formation of nanoparticles by electrostatic interactions between both functional groups.

A number of CH modifications have been proposed to enhance the efficacy of CH as a nucleic acid vector, namely the introduction of imidazole moieties into the CH backbone (CHimi) which has proven effective in promoting the escape of the nanoparticles from the endocytic pathway [10]. The partial quaternization (trimethylation) of CH gives origin to trimethylchitosan (TMC), which has fixed positive charges, being soluble at a wider pH range and exhibiting enhanced mucoadhesive potential [11].

CDX2, a transcription factor belonging to the *caudal*-related homeobox gene family, is a master regulator of intestinal cell survival and differentiation. Besides its involvement in the normal development of the intestine, it is also present in every foci of aberrant intestinal differentiation, such as intestinal metaplasia (IM) of the stomach, which is a precursor lesion of gastric cancer [12], [13]. It was shown that CDX2 regulates its own expression and is bound to its own promoter in mouse intestine and in human gastric IM, suggesting that a positive autoregulatory mechanism could be critical for the maintenance of the intestinal phenotype [14]. In colorectal cancer, there are multiple evidences that CDX2 has a tumor suppressor function [15], [16]. However, it was also recently described as a lineage-survival oncogene in this context [17], which might extend to other cancer types associated with intestinal differentiation. Thus, CDX2 appears as an obvious therapeutic target of premalignant lesions with aberrant intestinal differentiation, for which specific treatments are lacking, and might also constitute an adjuvant therapy in cancer.

In our study we used a nanoparticle delivering system of siRNA directed to CDX2, using CHimi and TMC as vectors, and showed that this system is able to downregulate CDX2 expression in gastric cell lines, and reaches the gastric mucosa in mouse gastric explants.

## Results and Discussion

With our study we intended first to assess the efficiency of CHimi and TMC as carriers of siRNA targeting CDX2 in gastric cell lines as a potential therapy to use in both IM and gastrointestinal cancers.

We used commercially available CH (MW 78 kDa) and TMC (MW 43 kDa) as starting material. Imidazole-grafted CH (CHimi) was synthesized with different degrees of substitution (DS) by amidation of the glucosamine residues, using a condensation system as previously described [10]. Polymers with 9% (CHimi 1) and 16% (CHimi 2) moles of imidazole moieties per mole of glucosamine residues were obtained (Table S1).

CHimi and TMC 0.1% (w/v) solutions were prepared in 5 mM acetate buffer (pH 5.5) and 20 mM HEPES buffered solution with 5% glucose (pH 7.4), respectively. The nanoparticles were then formed by spontaneous electrostatic interactions between CHimi or TMC solutions and a mixture of 3 siRNAs directed to different sequences in CDX2 (siCDX2).

**Figure 1:**
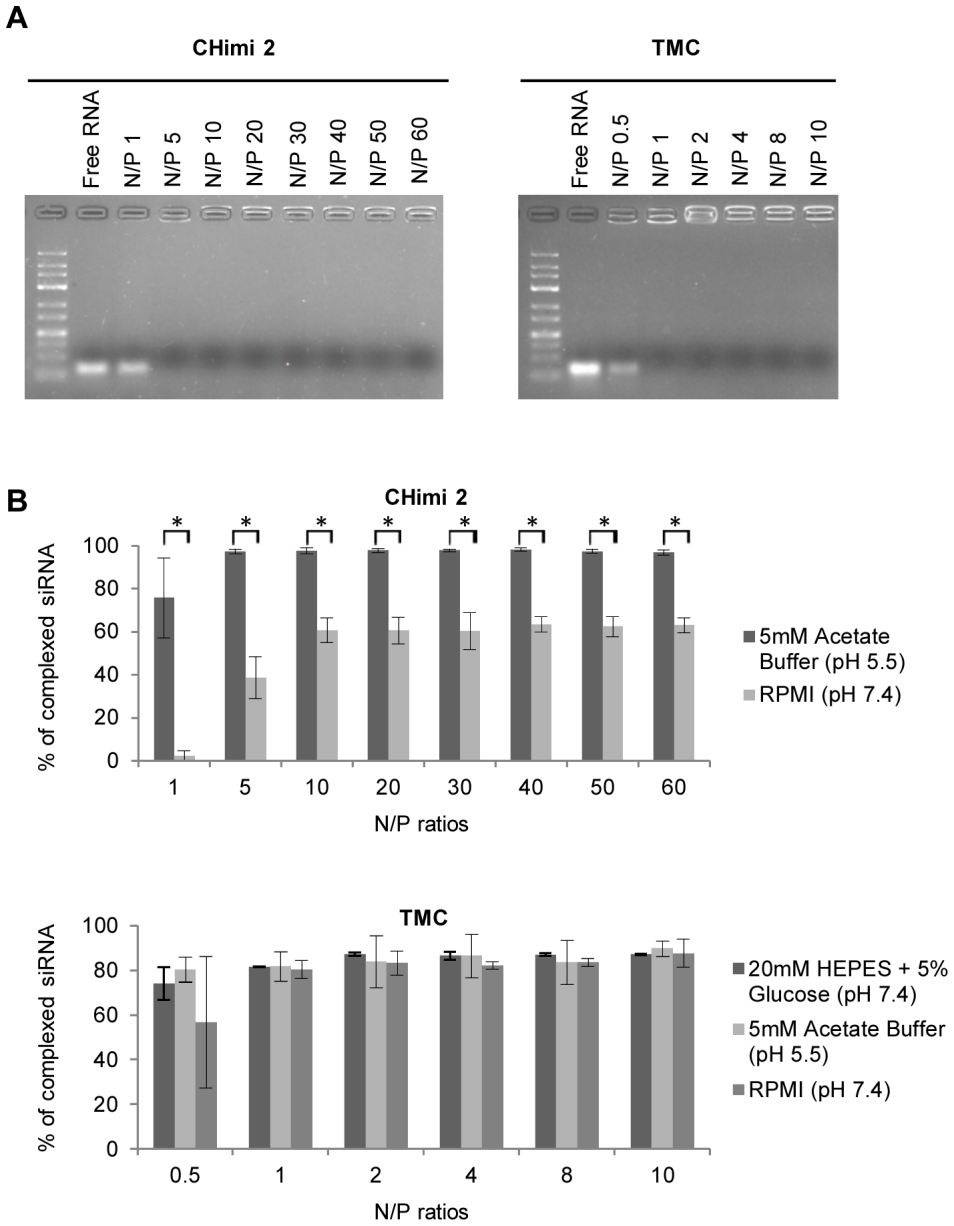
CHimi and TMC/siRNA nanoparticle complexation capacity. (**A**) Nanoparticle complexation capacity determined by detecting free siRNA migration in an agarose gel electrophoresis. Free siRNA was used as positive control. Nanoparticles with different N/P ratios were tested. (**B**) SYBRGold exclusion assay. The complexation capacity of the prepared nanoparticles was analysed at different N/P rations and at two different pHs (n = 3; average ± SD). * p<0.01.

To determine the amount of CHimi and TMC polymers required to complex the siRNA, nanoparticles with different N/P molar ratios were prepared (N/P ratios – the ratio of the moles of primary amines in CHimi or trimethylated amines in TMC to phosphate groups in siRNAs). Complexation of siRNA by the polymers was determined by detecting free siRNA in agarose gel electrophoresis, using different N/P ratios; free siRNA migrates towards the positive pole whereas complexed siRNA does not migrate. The results obtained showed that independently of the DS, CHimi halted siRNA mobility at N/P ratios >1, while TMC impaired migration at ratios >0.5 (Fig. 1A). The complexation capacity of the nanoparticles was further tested using a SYBRGold exclusion assay that corroborated the previous results, when incubated in the same buffers where they were prepared (Fig. 1B). Furthermore, the complexation of both systems was tested at pH 5.5 (acetate buffer) and in RPMI (Roswell Park Memorial Institute) media (physiologic pH), and the results showed that TMC particles were able to complex >80% of the siRNA at both pHs, while CHimi nanoparticles decreased the complexation capacity to around 60% at physiologic pH (Fig. 1B).

**Figure 2:**
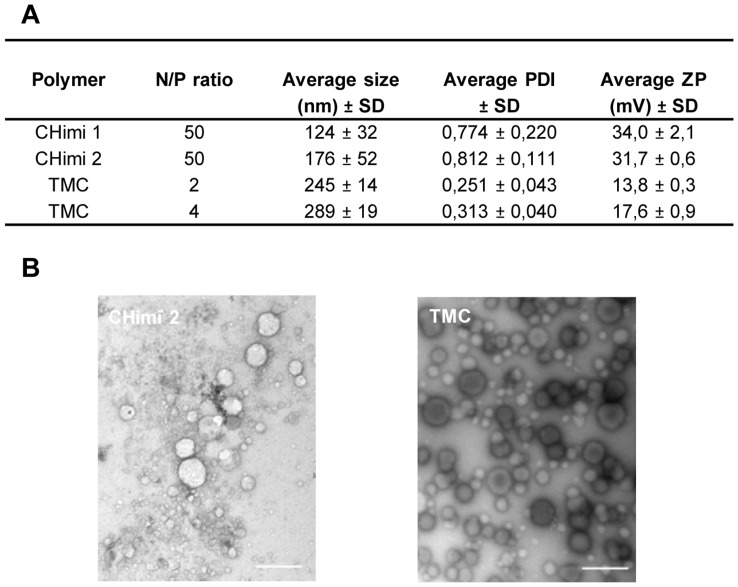
CHimi and TMC/siRNA nanoparticle characterization. (**A**) Size (hydrodynamic diameter), polydispersity index (PDI) and charge (ZP, zeta potential) of CHimi and TMC/siRNA nanoparticles determined using a Zetasizer Nano ZS at pH 5.5 and 7.4, respectively (n = 3; average ± SD). (**B**) Transmission electron microscopy images of CHimi2 and TMC nanoparticles. Scale bar 500 nm.

N/P ratios of 50 and of 2 or 4 were selected to further characterize the nanoparticles based on CHimi and TMC, respectively. Characterization of size (hydrodynamic diameter), polydispersity index (PDI), and particle net charge (zeta potential (ZP)) was performed using a Zetasizer Nano ZS (Fig. 2A). CHimi nanoparticles were smaller than the TMC ones (average size <180 and <290 nm, respectively), but more polydisperse. Both displayed a positive net charge. When evaluated in RPMI media, the charge of the CHimi nanoparticles significantly decreased while the net charge of the TMC ones remained in a similar range, as expected (Table S2). Further characterization of the nanoparticles was performed by transmission electron microscopy (TEM) analysis (Fig. 2B), which revealed that the nanoparticles had a nearly spherical shape and, as previously determined, were polydisperse.

We next examined the cellular uptake of the nanoparticle formulations by AGS and IPA220 cells (gastric carcinoma cell lines that constitutively express CDX2). FITC-labelled siRNA was used to assess the percentage of internalization by flow cytometry, 24 hours post-transfection. Our data shows that TMC nanoparticles were taken up more efficiently compared to the CHimi nanoparticles (Table S3), which might be explained by the fact that transfection is performed under physiological conditions (namely the pH) in which CHimi nanoparticles decrease their complexation capacity and tend to aggregate.

The effect of the nanoparticles on cell viability was assessed 48 hours post-transfection using a resazurin-based assay. The different formulations tested were found to be non-toxic, with cell viabilities above 80% (Fig. S1), which is an advantage over other delivery systems [18].

**Figure 3:**
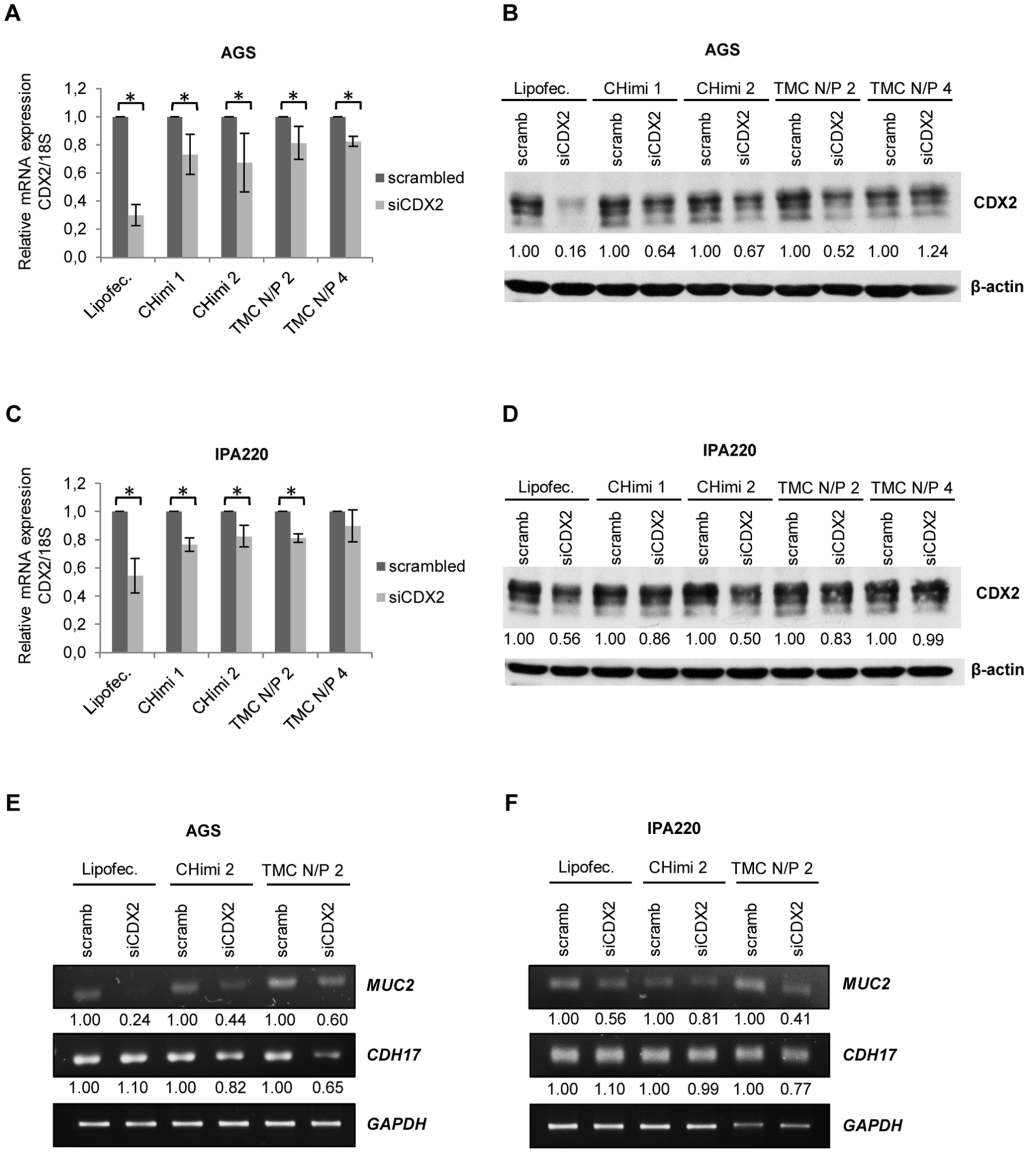
CHimi and TMC nanoparticle transfection assessments. (**A, C**) *CDX2* mRNA levels in AGS and IPA220 cells 48 hours post-transfection with 50 nM and 75 nM of scrambled and CDX2 siRNA, respectively (n = 3; average ± SD), * p<0.05. mRNA was quantified with real-time PCR and normalized to the corresponding *18S* rRNA level. (**B, D**) Western blots and respective quantification showing levels of CDX2 in AGS and IPA220 cells 48 hours post-transfection with 50 nM and 75 nM of scrambled and CDX2 siRNA, respectively. β-actin was used as loading control. (**E, F**) RT-PCR results and respective quantification showing expression of the CDX2 targets, MUC2 and CDH17, in AGS and IPA220 cells 48 hours post-transfection with 50 nM and 75 nM of scrambled and CDX2 siRNA, respectively. GAPDH was used as endogenous control. Lipofectamine/siRNA complexes were used as controls in all experiments.

To determine the functional capacity of the nanoparticles to downregulate a target mRNA, cells were transfected with siRNAs targeting *CDX2* (siCDX2) or scrambled siRNAs sequences as control (scramb) and lysed 48 hours later. *CDX2* mRNA levels were measured by quantitative real-time PCR and normalized to *18S* rRNA levels. Results showed a decrease in *CDX2* mRNA levels for all formulations tested and in both cell lines (Fig. 3A and 3C). To evaluate the effect on protein synthesis, cells were collected 48 hours post-transfection and CDX2 protein levels were assessed by polyacrylamide gel electrophoresis and Western blotting. When compared to cells transfected with scramb sequences, those transfected with the different nanoparticle formulations showed a clear reduction in CDX2 protein levels (Fig. 3B, 3D and Fig. S2). There were some discrepancies in the levels of CDX2 mRNA and protein downregulation. This can be attributed to the fact that siRNAs might not always lead to mRNA degradation but only impair translation [6]. We further showed that CDX2 downregulation had an impact on the expression of MUC2 and CDH17 (Fig 3E and 3F), known CDX2 targets.

Taken together, our results show that the tested nanoparticles, while displaying different properties and internalization efficiencies, exhibited similar efficacy in downregulating CDX2 in our *in vitro* model. This can be attributed to the effect of the imidazole moieties (in CHimi) in enhancing endosome escape and increasing the transfection efficiency. The combination of the two functionalities - imidazole rings and trimethylated amines - in the chitosan backbone is presently being explored to improve the transfection outcome mediated by this material.

One of the most striking differences between the two different compounds was their behaviour at different pHs, which is an extremely relevant topic when the aim is to obtain a localized delivery to the gastrointestinal mucosa. This route of administration is highly desirable, as it would improve the compliance and efficacy of the therapy, with reduced side effects. Both nanoparticles were stable at acidic pH and could be used to target the gastric mucosa.

In the gastrointestinal context, another important barrier that nanoparticles have to face is the mucus layer [19]. As stated previously, CH and its derivatives are mucoadhesive due to the electrostatic interactions with mucins. This characteristic has the advantage of prolonging the residence time of the nanoparticles in the mucus with the consequent increase in bioavailability [20], but this depends on the characteristics and turnover time of the local mucus layer [21]. In fact, the mucus system varies along the digestive tract; in stomach and in colon it is composed of two layers: an inner, dense, firmly attached mucus layer and an outer, unattached, loose mucus layer [22]. The inner one in colon is normally impermeable to bacteria and to beads of bacterial cell size, but in the stomach is not [23], [24]. So, it is likely that, in colon, rather than reaching the more slowly cleared inner layer, mucoadhesive nanoparticles might be trapped in the loosely adherent mucus layer and become vulnerable to rapid clearance.

**Figure 4:**
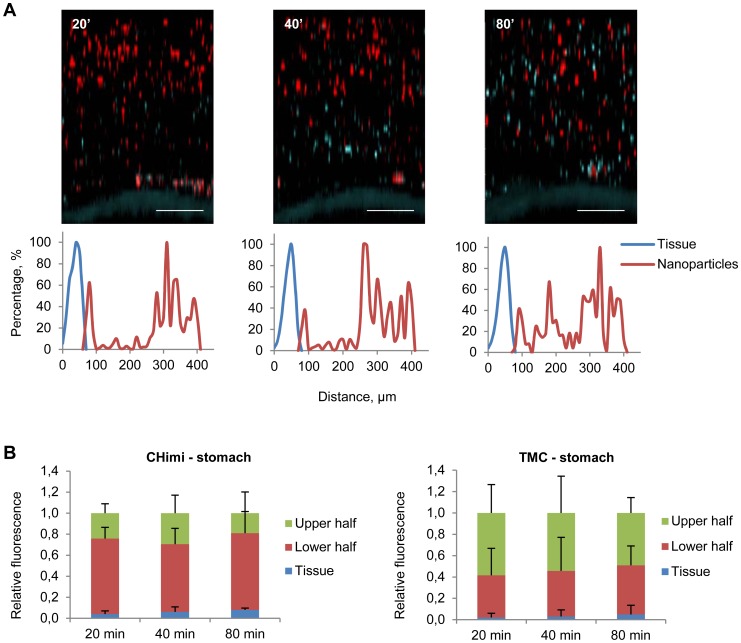
Mucus penetrability of nanoparticles in mouse gastric explants. (**A**) Representative Z – stack projections of TMC/siRNA nanoparticles in stomach explants and the corresponding normalised intensity plots; tissue is blue and nanoparticles are red. Scale bars 100 µm. (**B**) Percentage of the total fluorescence intensity of TMC and CHimi2/siRNA nanoparticles in each plan (tissue, lower half, and upper half) at each time point. Data are presented as means ± SD (n = 4).

**Figure 5:**
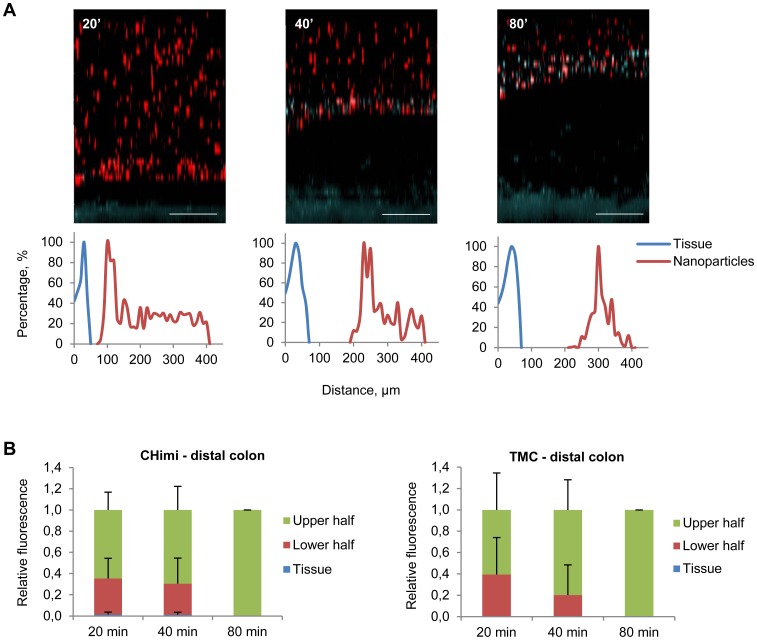
Mucus penetrability of nanoparticles in mouse distal colon explants. (**A**) Representative Z – stack projections of TMC/siRNA nanoparticles in distal colon explants and the corresponding normalised intensity plots; tissue is blue and nanoparticles are red. Scale bars 100 µm. (**B**) Percentage of the total fluorescence intensity of TMC and CHimi2/siRNA nanoparticles in each plan (tissue, lower half, and upper half) at each time point. Data are presented as means ± SD (n = 3).

Taking this into account we determined the behaviour of the nanoparticles in the gastrointestinal mucus, assessing whether these were trapped in the loosely or inner adherent mucus layers, or penetrated both layers and reached the underlying epithelium. To evaluate the capacity of nanoparticles to penetrate the mucus barrier, we used a method where explants from mouse stomach and colon were mounted in a horizontal perfusion chamber in which mucus was continuously secreted, with preservation of its biological properties [25]. The explants were allowed to secrete mucus for 20 min, after which nanoparticles with fluorescent-labelled siRNA were placed on the top and allowed to sediment into the mucus for another 20 min. Then, the position of nanoparticles relatively to the epithelium was evaluated by confocal microscopy (at 20, 40 and 80 min). CHimi and TMC nanoparticles were observed over the entire thickness of the gastric mucus at all evaluation time points, but were not found in the inner mucus layer in the distal colon, instead being pushed upwards as new mucus was being secreted (Fig. 4 and 5; Fig. S3). Nanoparticles were also able to penetrate the mucus of the small intestine and proximal colon (Fig. S4). A recent study detected chitosan nanoparticles at the surface of epithelial cells in small intestine and colon [26], partly contradicting our results. However the exact colonic location was not referred and the delivery methodology used might have disrupted the mucus barrier contributing to the observed results.

When comparing the performance of the two vectors, although both behaved similarly in both locations, CHimi seemed to have a higher capacity of reaching further down into the mucus layers. This can be attributed to a lower mucoadhesivity due to low charge density. With this method we reproduced the *in vivo* behaviour of the mucus system, which offers the opportunity to follow changes in mucus properties over time. This is a great advantage over other methods that use fixed mucus at given time points. In fact, a recent study suggested that stored or purified mucus exhibits significantly altered properties as compared to fresh mucus, namely increased hydrophobicity and stickiness which can hamper the anticipation of the nanoparticle behaviour *in vivo* [27].

As shown, this method is useful to study the penetrability capacity of drug carrier nanoparticles in real-time, anticipating their behaviour *in vivo* and allowing studies of the effect of variations in terms of charge, size or pH. Additional work is needed to determine whether other alterations of the CHimi and TMC nanoparticles, such as poly(ethylene glycol) tethering to the nanoparticle surface [28], can confer mucus penetrating properties in the distal colon, in order to use them in a wider range of gastrointestinal diseases.

In summary, the nanoparticles used in our study were able to downregulate the expression of CDX2 protein without affecting cell viability in our *in vitro* models. Furthermore, these nanoparticles were able to penetrate gastric mucus, but not distal colonic mucus, which is promising for their use as a gastric delivery system *in vivo*.

## Materials and Methods

### siRNAs

A mix of 3 validated *CDX2* siRNAs and 3 scrambled siRNAs (Sigma, St. Louis, MO) were used (sequences are shown in Table S4). A *CDX2* siRNA containing a Fluorescein isothyocianate-labeled strand (FITC) (Sigma) was used in the cellular uptake and mucus penetrability studies.

### Polymers

Technical grade chitosan (Chimarin, molecular weight 78 kDa, degree of acetylation 13%, apparent viscosity 8 mPas) was supplied by Medicarb, Sweden. CH was purified by filtration of a CH acidic solution and subsequent alkali precipitation (NaOH 1 M) and collected after freeze-drying. CH was posteriorly modified by amidation of a percentage of its glucosamine residues (CHimi) using an EDC/NHS condensation system as previously described [10]. Imidazole-4-acetic acid sodium salt (ImiAcOH), N-(3-dimethylaminopropyl)-N0-ethylcarbodiimide hydrochloride (EDC) 98% and N-hydroxysuccinimide (NHS) 97% were purchased from Sigma. Trimethylchitosan (KiOmedine-TMC, DA 8.45%, degree of quaternization – DQ – 28.82%) was purchased from KitoZyme SA, Herstal, Belgium. TMC was purified by ethanol precipitation (the polymer was dissolved in an aqueous solution at a final concentration of 0.5% (w/v), filtered through a Buchner funnel, precipitated with 1∶1 (v/v) ethanol/ether solution and collected after freeze-drying.

### Polymer characterization

CHimi polymers were characterized by Fourier transform infrared spectroscopy (FTIR) using the potassium bromide (KBr) technique. Each pellet was prepared by blending 2 mg of the polymer (vacuum dried 24 hours at 60°C) with 200 mg of KBr (dried 24 hours at 105°C). After a 5 min purge of the sample chamber with N_2_, the infrared spectra were immediately recorded in a FTIR system 2000 from Perkin-Elmer by accumulation of 200 interferograms at a 4 cm^−1^ spectral resolution. The degree of substitution of the glucosamine residues was calculated as previously described [10]. TMC molecular weight was characterized by gel permeation chromatography; measurements were performed in 0.33 M NaCH_3_COOH/0.28 M CH_3_COOH eluent at a flow rate of 1 mL.min^−1^ (MW 43.3 kDa). TMC degree of quaternization (DQ) was determined by ^1^H-Nuclear Magnetic Resonance (Bruker Avance II); samples were dissolved in D_2_O (2 mg.mL^−1^) at 60°C overnight; the DQ was calculated according to Mourya [29] (30.1%). Degree of acetylation was calculated by FTIR (11.1%).

### CHimi and TMC solution preparation

Polymers were vacuum dried overnight at 60°C. CHimi was diluted in acetic acid 1% overnight and posteriorly added 5 mM acetate buffer (pH 5.5), under stirring; pH was corrected to 5.5 by addition of NaOH 1 M. TMC was diluted in 20 mM HEPES buffered solution with 5% (w/v) glucose (pH 7.4), under stirring; pH was corrected to 7.4 by addition of NaOH 1 M. All solutions were prepared with a final concentration of 0.1% (w/v) in polymer.

### Nanoparticle preparation

CHimi- and TMC-siRNA nanoparticles were formed by mixing equal volumes of CHimi and TMC 0.1% solutions with siRNA (previously diluted in 5 mM acetate buffer pH 5.5 or 20 mM HEPES with 5% (w/v) glucose, respectively). Nanoparticles with different polymer to siRNA ratios were prepared (N/P, the ratio of the moles of primary amines in the case of CHimi or trimethylated amines in the case of TMC to moles of phosphate groups in siRNA).

### Nanoparticle characterization

Complexes were prepared using 10 µg of siRNA at various N/P molar ratios and diluted to 1 mL in acetate buffer pH 5.5 (CHimi) or HEPES Glucose pH 7.4 (TMC). Zeta potential, mean hydrodynamic size and polydispersity index of the complexes were determined using a Zetasizer Nano ZS (Malvern, UK). The Smoluchowski model was applied for zeta potential determination and cumulative analysis was used for mean particle size determination. All measurements were performed in triplicate, at 25°C. The morphology and size of the nanoparticles was also evaluated by transmission electron microscopy. A total of 10 µL of nanoparticle suspension was mounted in a 400 mesh carbon-coated nickel grid for 2 min, stained with 1% uranyl acetate and examined under a JEOL JEM-1400 transmission electron microscope (Tokyo, Japan). Images were digitally recorded using a Gatan SC 1000 ORIUS CCD camera (Warrendale, PA, USA).

### Nanoparticle complexation capacity

Nanoparticles were prepared at various N/P molar ratios as previously described. For the agarose gel electrophoresis, 0.3 µL of 100 µM siRNA were used for the preparation of the complexes in a final volume of 30 µL, and 20 µL of each complex solution together with 4 µL of loading buffer (Fermentas) were migrated on a 4% (w/v) agarose gel with RedSafe (CHEMBIO) in a 90 V field for 1 hour, using Tris-acetate-EDTA (pH 8) as the running buffer. siRNA complexation capacity was also determined by a SYBRGold exclusion assay. CH- or TMC-based nanoparticles were prepared as previously described and then incubated in 5 mM sodium acetate buffer (pH 5.5), 20 mM HEPES buffered saline solution with 5% (w/v) glucose (pH 7.4) or Roswell Park Memorial Institute (RPMI) 1640 media (pH 7.4) (Gibco, Invitrogen, Carlsbad, CA) in a black-walled 96-well plate at RT and then 2 µL of a 1∶100 SYBRGold (Invitrogen) solution (in TAE buffer) were added to each well (final volume of 200 µL). After 10 min fluorescence was measured (λ_exc_ = 485 nm, λ_em_ = 540 nm) using a microtiter plate reader (SynergyMx, Biotek). Results are given as the percentage of complexation, where 100% represents non- intercalating dye (the total amount of siRNA is complexed). Samples with the same mass ratio of polymer without siRNA were used as controls in order to subtract any background fluorescence originating from the polymers.

### Cell culture

The cell lines AGS (ATCC) and IPA220 [30] were cultured at 37°C and 5% CO2 and maintained in RPMI media supplemented with 10% (v/v) foetal bovine serum (FBS) (Gibco, Invitrogen) and 1% (v/v) antibiotics (10 U/mL penicillin and 10 U/mL streptomycin (Gibco, Invitrogen).

### Transfection

Cells were seeded 24 hours prior to transfection in 12-well tissue culture plates at a density of 1×10^5^ (AGS) or 2×10^5^ cells/well (IPA220). Two hours before transfection, cell culture medium was removed and replaced with un-supplemented fresh medium. Nanoparticles were prepared as previously described at a final concentration of siRNA of 50 nM (in AGS) or 75 nM (in IPA220) and added to the cells. Lipofectamine (Invitrogen) complexes were used as a positive control, according to the manufactureŕs instructions. Cells were incubated with the complexes for 24 hours, after which time the medium was substituted with complete fresh medium.

### Nanoparticle internalization

The cellular uptake of the nanoparticles was evaluated by transfecting cells using a FITC-siRNA. After 24 hours of incubation with the nanoparticles, cells were washed with 1% phosphate buffer saline (PBS), trypsinized, washed twice with PBS and resuspended in PBS with 2% (v/v) FBS and 1 mM EDTA. Cellular uptake was evaluated by fluorescent activating cell sorting (FACS) using a BD Calibur flow cytometer (Becton Dickinson, San Jose, CA). For each sample, 10000 events were counted. Non-transfected cells were used as negative controls and data was analysed using Flow Jo 9.6 software (TreeStar Inc).

### Nanoparticle toxicity

Cell viability was assessed using a resazurin based assay (Sigma). Viable cells reduce resazurin (a blue non-fluorescent compound) to resofurin (a red fluorescent product). As viable cells continuously convert resazurin to resofurin, an indirect quantitative measure of viability was obtained. Cells (1×10^5^ of AGS and 2×10^5^ of IPA220) were seeded into 96-well plates and transfected 24 hours later, as previously described. Fluorescence was measured (λ_exc_ = 530 nm; λ_em_ = 590 nm) using a microtiter plate reader (SynergyMx, Biotek).

### mRNA extraction and reverse transcriptase polymerase chain reaction (RT-PCR)

Cells were washed with PBS and treated with chitosanase (Merck, Darmstadt, DE), as previously described [31]. Total RNA was extracted using TRI Reagent (Sigma) and converted to cDNA using SuperScript II ReverseTranscriptase (Invitrogen). *CDX2* (5′-TTC ACT ACA GTC GCT ACA TCA CC-3′ and 5′- TTG TTG ATT TTC CTC TCC TTT GC -3′) and *18S* (5′- CGC GCG CTA GAG GTG AAA TTC -3′ and 5′- CAT TCT TGG CAA ATG CTT TCG -3′) were amplified with SYBR Green (Applied Biosystems, Foster City, CA) in an ABI Prism 7500 thermocycler. *18S* rRNA levels were used for normalization. *MUC2* (5′-GCC TGC AGA GCT ATT CAG AAT TC-3′ and 5′-ATC TTC TGC ATG TTC CCA AAC TC-3′), *CDH17* (5′-CGA AGG CTC AGT AAG GCA GAA-3′and 5′-CAT CCA GGT CTG TGG CAT TG-3′) and *GAPDH* (5′-TCA AGG CTG AGA ACG GGA AG-3′and 5′-AGA GGG GGC AGA GAT GAT GA-3′) cDNAs were amplified with SYBR Green using the following thermocycler program: enzyme activation step (1 cycle) of 10 min at 95°C; denaturation step of 15 sec at 95°C and annealing/extension step of 1 min at 60°C (32 cycles). These samples were ran in a 2% agarose gel and visualized in a Chemidoc XRS imaging system (BioRad, CA) equipped with a SYBR Green detection filter. *GAPDH* mRNA levels were used for normalization. Each experiment was carried out at least twice.

### Protein extraction and Western blot

Cell pellets were resuspended in RIPA buffer (50 mM Tris–HCl pH 7.4, 150 mM NaCl, 2 mM EDTA, 1% NP-40, 0.1% sodium dodecyl sulphate) in the presence of complete protease inhibitors cocktail (Roche, Indianopolis, IN). Quantification of total protein was determined by bicinchoninic acid (BCA) protein assay (Pierce, Rockford, IL). Total protein extracts (30–50 µg) were subjected to standard sodium dodecyl sulphate–polyacrylamide gel electrophoresis, transferred onto a nitrocellulose membrane (Amersham, GE Healthcare, UK) and incubated with primary antibodies overnight at 4°C: mouse monoclonal anti-CDX2 (1∶500, Biogenex) and goat polyclonal anti-β-actin (1∶8000, Santa Cruz Biotechnology) in 5% BSA in tris-buffered saline 0.01% Tween-20 (Sigma). Peroxidase-conjugated secondary antibodies (for CDX2 goat anti-mouse-HRP, 1∶2000 and for actin goat anti-rabbit-HRP, 1∶2000, both from Santa Cruz Biotechnology) were used and developed with the ECL detection kit (Amersham). Quantification of the western blots was performed using the Quantity One software (BioRad, CA). Each experiment was performed at least twice and a representative result is shown.

### Nanoparticle penetrability in gastrointestinal mucus

Animal experiments were approved by the Animal Ethics committee, University of Gothenburg. Gastric and colonic explants were obtained and mounted in an image chamber, as previously described [23]. Briefly, mice (C57/Bl6 males, 10–12 week old) were anesthetized with isofluorane and killed by cervical dislocation. The stomach and distal colon were dissected and flushed with ice-cold oxygenated Krebs' buffer, and kept on ice followed by opening along the mesenteric border and removal of the longitudinal muscle layer by blunt dissection. The specimen was subsequently mounted in an Ussing-like horizontal chamber for image acquisition (surface area 1.8 mm^2^ for colonic explants and 16.1 mm^2^ for gastric explants). The apical chamber was filled with 1.5 mL Krebs' mannitol buffer, and the serosal side was constantly perfused with Krebs' glucose buffer containing Calcein Violet Blue tissue staining (1 µl.mL^−1^ in the serosal perfusate; Invitrogen). The chamber was heated to 37°C and kept at a constant temperature during the whole experiment. The tissue was incubated for 20 min followed by removal of the majority of the apical solution. A suspension of CHimi or TMC and siRNA-FITC nanoparticles prepared as described above but diluted in Krebs' buffer was then added to the apical surface (final concentration of siRNA 100 µM, final volume 1.5 mL) and the nanoparticles were left to sediment into the mucus for 20 min. The distribution of the nanoparticles in the mucus was analyzed by confocal imaging using XY stacks (640×640 µm) in a LSM 700 Axio Examiner 2.1 confocal imaging system with a Plan-Apochromat x20/1.0DIC water objective (Zeiss) at 20, 40 and 80 min after addition of the nanoparticles. The optical thickness of the section was 2.8 µm, and the sections were taken in 10 µm intervals. Images were processed using the ZEN 2010 software (Zeiss). Mucus penetrability was analysed by quantification of fluorescence intensity in the different stacks.

### Statistics

Data are presented as means ± standard deviation (SD). Student's t-test was used when analysing differences between the groups. Results were considered statistically significant when p<0.05.

## Supporting Information

Figure S1 **Cell metabolic activity was assessed using a resazurin assay; fluorescence was measured 48 hours after transfection with CHimi/siRNA (**A**) and TMC/siRNA (**B**) nanoparticles (50 nM and 75 nM of siRNA in AGS and in IPA220 cells, respectively).** (n = 3; average ± SD).
(DOCX)

Figure S2 **Quantification of western blots showing CDX2 protein expression changes in AGS (**A**) and IPA220 (**B**) 48 hours post-transfection with 50 nM and 75 nM of scrambled and CDX2 siRNA, respectively.** β-actin was used as loading control (n = 3; average ± SD) * p<0.05.
(DOCX)

Figure S3 (**A**) **Representative Z – stack projections of CHimi2/siRNA nanoparticles in stomach explants and the corresponding normalised intensity plots; tissue is blue and nanoparticles are red.** Scale bars 100 µm. (**B**) Representative Z – stack projections of CHimi2/siRNA nanoparticles in distal colon explants and the corresponding normalised intensity plots; tissue is blue and nanoparticles are red. Scale bars 100 µm.
(DOCX)

Figure S4 (**A**) **Representative Z – stack projections of TMC/siRNA nanoparticles in small intestine explants, 80 min post-administration; tissue is blue and nanoparticles are red.** Scale bars 100 µm. (**B**) Representative Z – stack projections of CHimi2/siRNA nanoparticles in proximal colon explants, 80 min post-administration; tissue is blue and nanoparticles are red. Scale bars 100 µm.
(DOCX)

Table S1 **Degree of imidazole substitution of the modified polymers, as determined by FTIR.**
(DOCX)

Table S2 **Size (hydrodynamic diameter), polydispersity index (PDI) and charge (ZP, zeta potential) of CHimi2 and TMC/siRNA nanoparticles determined using a Zetasizer Nano ZS at pH 7.4 (n = 3; average ± SD).**
(DOCX)

Table S3 **Internalization of nanoparticles assessed by flow citometry using FITC-labelled siRNA, 24 hours after transfection (n = 3; average ± SD).**
(DOCX)

Table S4 **Sequences of the siRNAs.**
(DOCX)
