# Supplementary material for: Modified-Chitosan/siRNA Nanoparticles Downregulate Cellular CDX2 Expression and Cross the Gastric Mucus Barrier
Source: PLoS One. 2014 Jun 12;9(6):e99449. doi: 10.1371/journal.pone.0099449 (PMC4055692; doi:10.1371/journal.pone.0099449)
Supplement: Figure S1 — Cell metabolic activity was assessed using a resazurin assay; fluorescence was measured 48 hours after transfection with CHimi/siRNA (A) and TMC/siRNA (B) nanoparticles (50 nM and 75 nM of siRNA in AGS and in IPA220 cells, respectively). (n = 3; average ± SD). (DOCX) [file pone.0099449.s001.docx]

**A**

Relative cell metabolic activity

**B**

Relative cell metabolic activity

**Figure S1.** Cell metabolic activity was assessed using a resazurin assay; fluorescence was measured 48 hours after transfection with CHimi/siRNA (**A**) and TMC/siRNA (**B**) nanoparticles (50 nM and 75 nM of siRNA in AGS and in IPA220 cells, respectively). (n=3; average ± SD).
