# Supplementary material for: Modified-Chitosan/siRNA Nanoparticles Downregulate Cellular CDX2 Expression and Cross the Gastric Mucus Barrier
Source: PLoS One. 2014 Jun 12;9(6):e99449. doi: 10.1371/journal.pone.0099449 (PMC4055692; doi:10.1371/journal.pone.0099449)
Supplement: Figure S2 — Quantification of western blots showing CDX2 protein expression changes in AGS (A) and IPA220 (B) 48 hours post-transfection with 50 nM and 75 nM of scrambled and CDX2 siRNA, respectively. β-actin was used as loading control (n = 3; average ± SD) * p<0.05. (DOCX) [file pone.0099449.s002.docx]

**A**

*

*

*

*

**B**

*

*

**Figure S2.** Quantification of western blots showing CDX2 protein expression changes in AGS **(A)** and IPA220 **(B)** 48 hours post-transfection with 50 nM and 75 nM of scrambled and CDX2 siRNA, respectively. β-actin was used as loading control (n=3; average ± SD) * p < 0.05.
