# Supplementary material for: Modified-Chitosan/siRNA Nanoparticles Downregulate Cellular CDX2 Expression and Cross the Gastric Mucus Barrier
Source: PLoS One. 2014 Jun 12;9(6):e99449. doi: 10.1371/journal.pone.0099449 (PMC4055692; doi:10.1371/journal.pone.0099449)
Supplement: Figure S4 — (A) Representative Z – stack projections of TMC/siRNA nanoparticles in small intestine explants, 80 min post-administration; tissue is blue and nanoparticles are red. Scale bars 100 µm. (B) Representative Z – stack projections of CHimi2/siRNA nanoparticles in proximal colon explants, 80 min post-administration; tissue is blue and nanoparticles are red. Scale bars 100 µm. (DOCX) [file pone.0099449.s004.docx]

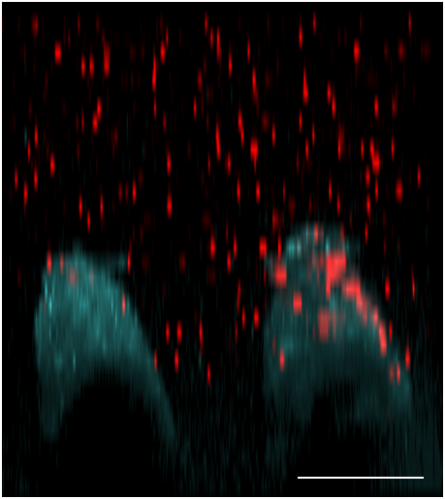

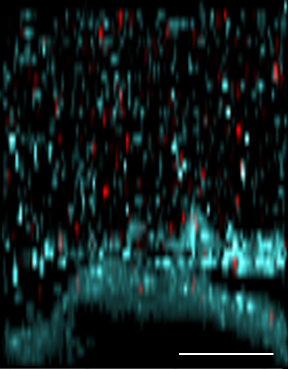


**A**

**B**

**Figure S4.** (**A**) Representative Z – stack projections of TMC/siRNA nanoparticles in small intestine explants, 80 min post-administration; tissue is blue and nanoparticles are red. Scale bars 100 µm. (**B**) Representative Z – stack projections of CHimi2/siRNA nanoparticles in proximal colon explants, 80 min post-administration; tissue is blue and nanoparticles are red. Scale bars 100 µm.
