# Supplementary material for: Modified-Chitosan/siRNA Nanoparticles Downregulate Cellular CDX2 Expression and Cross the Gastric Mucus Barrier
Source: PLoS One. 2014 Jun 12;9(6):e99449. doi: 10.1371/journal.pone.0099449 (PMC4055692; doi:10.1371/journal.pone.0099449)
Supplement: Table S1 — Degree of imidazole substitution of the modified polymers, as determined by FTIR. (DOCX) [file pone.0099449.s005.docx]

**Table S1.** Degree of imidazole substitution of the modified polymers, as determined by FTIR.

| **POLYMER** | **DS theoretical (%)** | **DS calculated by FTIR (%)** |
| --- | --- | --- |
| CH | ------ | ------ |
| CHimi 1 | 10% | 9 |
| CHimi 2 | 20% | 16 |
