# Supplementary material for: Modified-Chitosan/siRNA Nanoparticles Downregulate Cellular CDX2 Expression and Cross the Gastric Mucus Barrier
Source: PLoS One. 2014 Jun 12;9(6):e99449. doi: 10.1371/journal.pone.0099449 (PMC4055692; doi:10.1371/journal.pone.0099449)
Supplement: Table S2 — Size (hydrodynamic diameter), polydispersity index (PDI) and charge (ZP, zeta potential) of CHimi2 and TMC/siRNA nanoparticles determined using a Zetasizer Nano ZS at pH 7.4 (n = 3; average ± SD). (DOCX) [file pone.0099449.s006.docx]

**Table S2.** Size (hydrodynamic diameter), polydispersity index (PDI) and charge (ZP, zeta potential) of CHimi2 and TMC/siRNA nanoparticles determined using a Zetasizer Nano ZS at pH 7.4 (n=3; average ± SD).

| **Polymer** | **N/P ratio** | **Average size (nm) ± SD** | **Average PDI ± SD** | **Average ZP (mV) ± SD** |
| --- | --- | --- | --- | --- |
| CHimi 2 | 50 | 530 ± 37 | 0.881 ± 0.179 | 6.6 ± 1.4 |
| TMC | 2 | 269 ± 47 | 0.386 ± 0.049 | 11.2 ± 0.2 |
