# Supplementary material for: Modified-Chitosan/siRNA Nanoparticles Downregulate Cellular CDX2 Expression and Cross the Gastric Mucus Barrier
Source: PLoS One. 2014 Jun 12;9(6):e99449. doi: 10.1371/journal.pone.0099449 (PMC4055692; doi:10.1371/journal.pone.0099449)
Supplement: Table S3 — Internalization of nanoparticles assessed by flow citometry using FITC-labelled siRNA, 24 hours after transfection (n = 3; average ± SD). (DOCX) [file pone.0099449.s007.docx]

**Table S3.** Internalization of nanoparticles assessed by flow citometry using FITC-labelled siRNA, 24 hours after transfection (n=3; average ± SD).

| **Sample** | **N/P ratio** | **AGS**  **% of positive cells** | **IPA**  **% of positive cells** |
| --- | --- | --- | --- |
| **WT** | **---** | 0.5 ± 0.3 | 0.6 ± 0.5 |
| **Lipofectamine®** | **---** | 73.6 ± 23.1 | 84.7 ± 4.5 |
| **CHimi 1** | **50** | 6.1 ± 3.9 | 4.4 ± 3.8 |
| **CHimi 2** | **50** | 3.3 ± 1.7 | 6.1 ± 4.2 |
| **TMC** | **2** | 99.0 ± 0.7 | 64.8 ± 8.0 |
| **TMC** | **4** | 97.9 ± 1.9 | 76.0 ± 10.2 |
