# Supplementary material for: Modified-Chitosan/siRNA Nanoparticles Downregulate Cellular CDX2 Expression and Cross the Gastric Mucus Barrier
Source: PLoS One. 2014 Jun 12;9(6):e99449. doi: 10.1371/journal.pone.0099449 (PMC4055692; doi:10.1371/journal.pone.0099449)
Supplement: Table S4 — Sequences of the siRNAs. (DOCX) [file pone.0099449.s008.docx]

**Table S4**. Sequences of the siRNAs.

| **siRNA** | | **Sequence 5’→ 3’** |
| --- | --- | --- |
| **CDX2** | **1** | **S**: GAC AAG GAC GUG AGC AUG UAC CCU A |
|  |  | **AS:** UAG GGU ACA UGC UCA CGU CCU UGU C |
|  | **2** | **S:** CUC CGG GAG GAC UGG AAU GGC UAC |
|  |  | **AS:** CUU CCG CAU CCA CUC GCA CAG GUU |
|  | **3** | **S:** AAC CUG UGC GAG UGG AUG CGG AAG |
|  |  | **AS:** CUU CCG CAU CCA CUC GCA CAG GUU |
| **scrambled** | **1** | **S:** GCC GAA AUG GCG ACG UCC AGA AUA U |
|  |  | **AS:** AUA UUC UGG ACG UCG CCA UUU CGG C |
|  | **2** | **S:** GCG CAG CGA AGU CGG UCG AUA CGU |
|  |  | **AS:** ACG UAU CGA CCG ACU UCG CUG CGC |
|  | **3** | **S:** GAU GCG GAC GGA GGA UCG AUG UCA |
|  |  | **AS:** UGA CAU CGA UCC UCC GUC CGC AUC |
